# The triglyceride-glucose index and neutrophil-to-lymphocyte ratio jointly predict the no-reflow phenomenon in T2DM patients with STEMI after primary PCI

**DOI:** 10.1371/journal.pone.0345466

**Published:** 2026-03-20

**Authors:** Jingyan Yang, Dongling Xu, Xiaobo Liu, Zixiong Zhao, Juan Zhang

**Affiliations:** 1 Department of Pathology, The Second Qilu Hospital of Shandong University, Jinan, Shandong, China; 2 Department of Cardiology, The Second Qilu Hospital of Shandong University, Jinan, Shandong, China; 3 Shandong Blood Center, Jinan, Shandong Province, China; 4 The Second Clinical School, Shandong University, Jinan, Shandong, China; University of Diyala College of Medicine, IRAQ

## Abstract

**Background:**

Patients suffering from ST-segment elevation myocardial infarction (STEMI) and type 2 diabetes mellitus (T2DM) face an elevated risk of the no-reflow phenomenon even after successful primary percutaneous coronary intervention (PPCI). This study aimed to develop an integrated predictive model combining the triglyceride-glucose (TyG) index and the neutrophil-to-lymphocyte ratio (NLR) for no-reflow in this high-risk population.

**Methods:**

A retrospective cohort of 524 patients with T2DM and STEMI undergoing PPCI was analyzed. No-reflow was defined as post-procedural TIMI flow grade ≤2. Multivariable logistic regression and receiver operating characteristic (ROC) curve analyses were employed.

**Results:**

The incidence of no-reflow was 8.97% (47/524). Both TyG index (adjusted odds ratio [aOR] 2.98) and NLR (aOR 1.23) were identified as independent predictors. Patients were stratified into four groups based on the optimal cut-offs for NLR (2.831) and TyG (9.347). The group with high levels of both markers had a substantially higher no-reflow incidence (23.21%) compared to the low-risk group (1.49%). The combined model (TyG + NLR) demonstrated superior predictive performance (AUC 0.785) over models containing either marker alone or baseline clinical factors.

**Conclusion:**

The combination of TyG index and NLR effectively stratifies the risk of no-reflow in T2DM-STEMI patients, potentially aiding the early identification of patients in need of targeted management.

## Introduction

Patients with ST-segment elevation myocardial infarction (STEMI) and concomitant type 2 diabetes (T2DM) remain at high risk for poor outcomes even after successful primary percutaneous coronary intervention (PPCI) [1]. Despite the efficacy of PPCI in restoring epicardial flow, the no-reflow phenomenon occurs more frequently in these patients, resulting in limitations to myocardial salvage, increased infarct size, and a deterioration in clinical prognosis [2,3]. The heightened susceptibility to no-reflow in diabetes is driven by a pathophysiological milieu characterized by persistent inflammation and significant metabolic dysregulation [4]. These processes collectively promote endothelial dysfunction, microvascular impairment, and aberrant myocardial metabolism [5–7]. This interplay between inflammation and metabolism provides a strong rationale for evaluating biomarkers that reflect these distinct yet interconnected pathways.

Inflammatory biomarkers, particularly the neutrophil-to-lymphocyte ratio (NLR), have consistently demonstrated predictive value for no-reflow and adverse outcomes in acute coronary syndromes [8,9]. Similarly, the triglyceride-glucose index (TyG), a recognized marker of insulin resistance (IR), has been associated with increased cardiovascular risk in STEMI patients undergoing PPCI [10,11]. However, extant research has predominantly evaluated inflammatory and metabolic biomarkers in isolation. The question of whether a combined approach that integrates both pathophysiological pathways would offer superior predictive power for no-reflow, specifically in this high-risk diabetic STEMI population, remains unanswered.

Consequently, we hypothesized that an integrated model combining NLR and the TyG index will more effectively stratify the risk of no-reflow in T2DM patients with STEMI than models based on either marker alone. This study aims to develop and validate such a model, which could facilitate earlier identification of high-risk patients and guide targeted therapeutic strategies.

## Subjects and methods

### Study design and participants

This retrospective cohort study enrolled patients with acute STEMI who were treated with PPCI at the Second Hospital of Shandong University between June 2018 and December 2024. Eligible patients met established STEMI diagnostic criteria and underwent PPCI within 12 hours of symptom onset [12]. From an initial screening of 1938 STEMI patients, we identified 598 with pre-existing type 2 diabetes mellitus (T2DM). Patients with a history of prior myocardial infarction (MI) or coronary revascularization (percutaneous coronary intervention [PCI] or coronary artery bypass grafting [CABG]) were not excluded; both first-presentation and recurrent STEMI patients were included in this analysis.

We applied the following exclusion criteria: (1) delayed PPCI (>12 hours after symptom onset) or procedures without stent implantation; (2) rescue PCI following unsuccessful thrombolysis; (3) cardiogenic shock; (4) acute pulmonary edema; (5) ventricular septal rupture; (6) cardiac tamponade; (7) severe respiratory, renal, or hepatic impairment; (8) active inflammatory or malignant conditions; (9) corticosteroid or immunosuppressant use within 48 hours before admission; and (10) missing essential clinical data.

**Fig 1:**
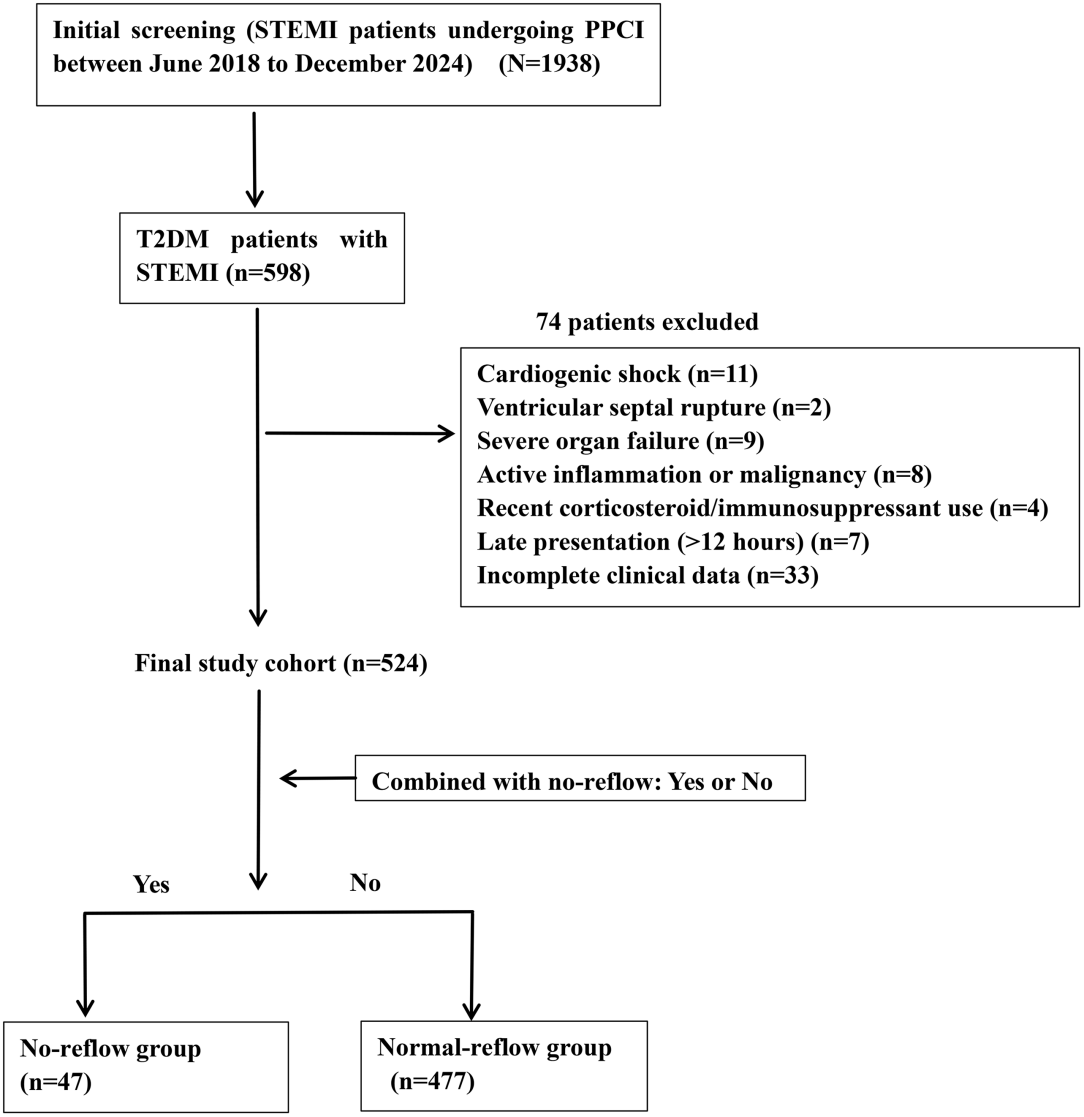
Patient flowchart. STEMI, acute ST-segment elevation myocardial infarction; PPCI, primary percutaneous coronary intervention; T2DM, type 2 diabetes mellitus.

Following these exclusions, 524 patients were included in the final analysis. We stratified participants according to intraoperative Thrombolysis in Myocardial Infarction (TIMI) flow grade into two groups: no-reflow (n = 47, 8.97%) and normal flow (n = 477, 91.03%). The research team responsible for data collection remained unaware of group assignments throughout the study. Fig 1 illustrates the patient selection process.

The present study adopts a retrospective cohort study design. Given the retrospective nature of the study, informed consent was waived by the Institutional Review Board of The Second Hospital of Shandong University (Approval No. KYLL-2025460), and and the requirement for individual patient consent was waived due to the retrospective nature of the study. All patient data were anonymized and handled in accordance with the ethical standards of the institution and the national research committee.

### PPCI and angiographic analysis

Upon admission, all patients received standard loading doses of antiplatelet agents and statin therapy, consisting of 300 mg aspirin, 300 mg clopidogrel or 180 mg ticagrelor, and 40 mg atorvastatin. Systemic anticoagulation was achieved with intravenous unfractionated heparin (70 U/kg) administered prior to coronary angiography and PPCI. Procedures were preferentially performed via radial access using conventional techniques. Following right radial artery catheterization, an appropriate guiding catheter was positioned to enable stent deployment. Stent selection and thrombus aspiration were performed at the operator’s discretion. Infarct-related artery flow was assessed immediately after stent implantation using the TIMI grading system. No-reflow was defined as TIMI flow grade ≤2, and TIMI flow grade 3 was classified as normal reperfusion [13]. The glycoprotein IIb/IIIa inhibitor tirofiban was available for intraprocedural administration when clinically indicated. Balloon predilation or postdilation was performed based on procedural requirements. All angiographic outcomes were independently reviewed by two experienced interventional cardiologists. Discrepancies in interpretation were resolved through consensus review. This standardized approach ensured consistent procedural management throughout the study cohort.

### Clinical data collection and definitions

The final dataset for analysis was accessed and locked on August 30, 2025. Data were collected retrospectively from the electronic medical records. Baseline clinical characteristics included demographic information (sex and age), cardiovascular risk factors (smoking status, hypertension, and dyslipidemia), and prior coronary artery disease (CAD) history (prior MI, PCI, or CABG). Laboratory parameters analyzed consisted of complete blood count tests, random plasma glucose, and lipid profiles (including triglycerides, TG), all obtained from blood samples collected during the initial emergency department visit, prior to any glucose-containing infusions and prior to primary PCI. Pre-admission medications, including antiplatelet agents, statins, ACE inhibitors/ARBs, beta-blockers, and glucose-lowering drugs, were also recorded. Inflammatory markers such as C-reactive protein (CRP) were assessed, along with routine coagulation parameters and cardiac injury biomarkers. The NLR was determined by dividing absolute neutrophil count by lymphocyte count [14]. The triglyceride-glucose (TyG) index was calculated using the formula: ln [TG (mg/dL) × glucose (mg/dL)/2] [15].

### Statistical analysis

All statistical analyses were performed using SPSS version 25.0 (IBM Corp., Armonk, NY, USA) and MedCalc version 20.0 (MedCalc Software Ltd., Ostend, Belgium). A two-tailed *P*-value <0.05 was considered statistically significant. Categorical variables are expressed as percentages and were compared using the Chi-square test. Normally distributed continuous variables are presented as mean±standard deviation and were analyzed with the independent samples t-test. Non-normally distributed continuous variables are reported as median with interquartile range and were compared using the Mann–Whitney U test. Multivariable logistic regression was employed to identify independent risk factors for the no-reflow phenomenon following primary percutaneous coronary intervention. To assess model stability and address potential overfitting given the number of events, bootstrap internal validation with 1,000 resamples was performed using MedCalc software. Bias-corrected and accelerated (BCa) 95% confidence intervals were calculated for the area under the curve (AUC). Subgroup analyses were performed stratified by sex, age (<60 vs. ≥ 60 years), and BMI (<25 vs. ≥ 25 kg/m²) using multivariable logistic regression adjusted for age and time-to-PCI. Interaction effects were assessed by including subgroup × biomarker interaction terms in the overall population models, with *P* for interaction derived from likelihood ratio tests. The independence between NLR and TyG was assessed using Pearson correlation analysis. Receiver operating characteristic (ROC) curve analysis was conducted to evaluate the predictive performance of relevant parameters for no-reflow and to determine optimal cutoff values. The DeLong test was used to compare the AUCs of the nested models.

## Results

### Baseline characteristics of study participants

Table 1 presents the baseline characteristics of the 524 participants stratified by post-procedural coronary flow status. The no-reflow phenomenon was observed in 47 patients (8.97%). Patients in the no-reflow group were significantly older than those in the normal-flow group (64.96 ± 10.56 vs. 60.43 ± 11.72 years, *P* = 0.011) and experienced a considerably longer median pain-to-balloon time (9.00 [IQR 5.50–12.00] vs. 6.00 [IQR 4.00–9.00] hours, *P* < 0.001). Furthermore, the proportion of smokers (including both current and former smokers) was higher among no-reflow patients (51.06% vs. 33.54%, *P* = 0.016). No significant differences were observed between groups in sex, hypertension, hyperlipidemia, prior CAD history, or pre-admission medications (all *P* > 0.05). The distribution of the infarct-related artery differed significantly between the normal reflow and no-reflow groups (*P* = 0.012). This disparity was primarily driven by a higher prevalence of left circumflex (LCX) (12.77% vs. 4.82%) and left main (LM) (4.26% vs. 0.42%) lesions among patients who developed no-reflow. The no-reflow phenotype was associated with a more pronounced inflammatory and metabolic profile. These patients showed markedly elevated levels of inflammatory markers (leukocytes, neutrophils, NLR, CRP) and metabolic parameters (TG, glucose, TyG index, glycated hemoglobin (HbA1c)) compared to the normal-flow group (all *P* < 0.05).

**Table 1:** Baseline characteristics of participants.

Characteristics	Total (n = 524)	Normal-reflow group (n = 477)	No-reflow group (n = 47)	*P*-value
Age, years	60.84 ± 11.69	60.43 ± 11.72	64.96 ± 10.56	**0.011**
Male, n(%)	387 (73.85)	353 (74.00)	34 (72.34)	0.804
Hypertension, n(%)	298 (56.87)	272 (57.02)	26 (55.32)	0.822
Hyperlipidemia, n(%)	132 (25.19)	118 (24.74)	14 (29.79)	0.447
Smoking, n(%)	184 (35.11)	160 (33.54)	24 (51.06)	**0.016**
**Hypoglycemic drugs, n(%)**				
Metformin	212 (40.46)	193 (40.46)	19 (40.43)	0.996
SLG-2 inhibitor	80 (15.27)	73 (15.30)	7 (14.89)	0.941
Insulin	146 (27.86)	129 (27.04)	17 (36.17)	0.183
Else	185 (35.31)	166 (34.80)	19 (40.43)	0.441
**Procedural Characteristics**				
Pain-to-balloon (hour)	5.59 ± 2.88	5.47 ± 2.79	7.20 ± 3.51	**<.001**
**Infarction related artery, n(%)**				
LAD	400 (76.34)	368 (77.15)	32 (68.09)	0.170
LCX	29 (5.53)	23 (4.82)	6 (12.77)	**0.016**
LM	4 (0.76)	2 (0.42)	2 (4.26)	**0.044**
RCA	91 (17.37)	84 (17.61)	7 (14.89)	0.639
**Laboratory Parameters**				
RBC, 10¹²/L	4.56 ± 0.56	4.55 ± 0.56	4.74 ± 0.50	**0.023**
Leukocytes, 10⁹/L	8.25 (6.82, 9.98)	8.08 (6.73, 9.96)	8.84 (8.09, 10.43)	**0.002**
Neutrophils, 10⁹/L	5.61 (4.46, 7.12)	5.46 (4.34, 7.05)	6.42 (5.67, 7.91)	**<.001**
Lymphocytes, 10⁹/L	1.67 (1.38, 2.06)	1.67 (1.38, 2.10)	1.66 (1.38, 1.89)	0.313
NLR	3.23 (2.44, 4.42)	3.20 (2.38, 4.36)	3.92 (3.05, 5.51)	**<.001**
CRP, mg/L	11.84 (7.99, 24.92)	11.45 (7.63, 24.20)	14.80 (10.14, 39.79)	**0.021**
D-dimer, ug/ml	0.59 (0.33, 1.03)	0.55 (0.33, 1.00)	0.95 (0.36, 1.25)	0.074
TG, mmol/L	1.42 (1.03, 2.01)	1.39 (1.02, 1.98)	1.85 (1.29, 2.50)	**<.001**
TC, mmol/L	3.98 (3.45, 4.64)	3.96 (3.45, 4.64)	4.09 (3.60, 4.60)	0.396
LDL, mmol/L	2.29 (1.74, 2.79)	2.28 (1.74, 2.79)	2.33 (1.69, 2.75)	0.902
HDL, mmol/L	1.15 (0.97, 1.38)	1.16 (0.98, 1.41)	1.15 (0.87, 1.34)	0.134
Glucose, mmol/L	7.95 (6.68, 9.48)	7.85 (6.60, 9.39)	8.90 (7.83, 11.02)	**0.005**
TyG index	9.15 ± 0.57	9.12 ± 0.56	9.51 ± 0.52	**<.001**
HbA1c,%	6.90 (6.50, 7.80)	6.90 (6.50, 7.70)	7.30 (6.90, 8.60)	**<.001**
Cre, μmol/L	70.00 (62.00, 79.00)	70.00 (62.00, 79.00)	70.00 (63.00, 79.00)	0.908
eGFR, ml/min/1.73 m^2^	90.09 ± 21.22	90.04 ± 21.22	90.63 ± 21.48	0.855

Data are presented as mean ± standard deviation, median [interquartile range], or n (%). *P*-values for continuous variables were derived from the Independent Samples t-test (for normally distributed data, presented as mean ± SD) or the Mann-Whitney U test (for non-normally distributed data, presented as median [IQR]). Categorical variables were compared using the Chi-square test. Abbreviations: LAD, left anterior descending artery; LCX, left circumflex artery; LM, left main coronary artery; RCA, right coronary artery; NLR, neutrophil-to-lymphocyte ratio; CRP, C-reactive protein; TG, triglyceride; TC, total cholesterol; LDL, low-density lipoprotein cholesterol; HDL, high-density lipoprotein cholesterol; TyG, triglyceride-glucose; HbA1c, glycated hemoglobin; Cre, creatinine; eGFR, estimated glomerular filtration rate.

### TyG and NLR as independent predictors of no-reflow

**Fig 2:**
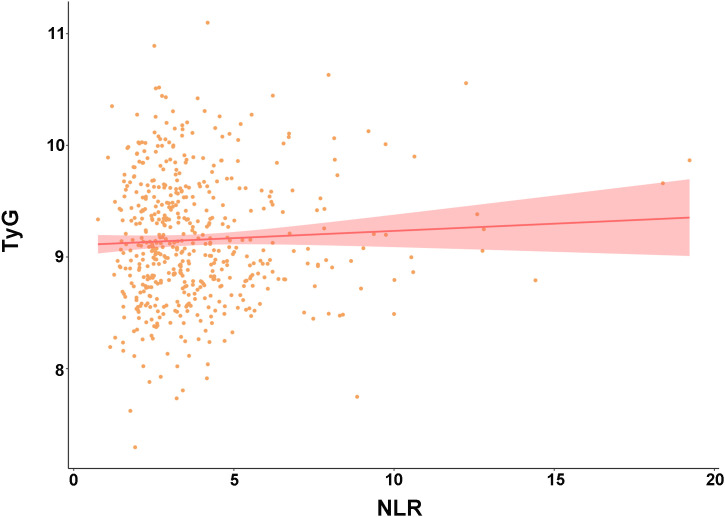
Scatter plot analyzing the correlation between TyG and NLR. Pearson correlation analysis reveals a negligible and non-significant positive linear association between the two biomarkers (r = 0.05, 95% CI: −0.04 to 0.14, *P* = 0.25). The absence of substantial collinearity justifies their simultaneous entry into the multivariable logistic regression model as independent predictors. NLR, neutrophil-to-lymphocyte ratio; TyG, triglyceride-glucose.

As shown in Table 2, univariate analysis identified several significant predictors for the no-reflow phenomenon, among which the TyG index (OR 3.36, 95% CI 1.96–5.76, *P* < 0.001) and NLR (OR 1.21, 95% CI 1.10–1.35, *P* < 0.001) demonstrated particularly strong associations. These two markers remained statistically significant in the multivariable analysis, with the TyG index emerging as the strongest independent predictor (adjusted OR 2.98, 95% CI 1.69–5.27, *P* < 0.001), followed by NLR (adjusted OR 1.23, 95% CI 1.09–1.39, *P* < 0.001). Pearson correlation analysis revealed no significant collinearity between TyG and NLR (r = 0.050, *P* = 0.250) (Fig 2). Given their robust performance in both univariate and multivariable analyses, TyG index and NLR were selected for further investigation in subsequent stratified analyses. The final multivariable model also confirmed pain-to-balloon time, age, red blood cell count, and CRP as independent predictors of no-reflow. Bootstrap validation with 1,000 resamples confirmed model stability.

**Table 2:** Univariable and multivariable logistic regression analysis for no-reflow.

Variables	Univariate		multivariate	
	OR (95%CI)	*P-*value	OR (95%CI)	*P-*value
Age	1.04 (1.01 ~ 1.06)	**0.012**	1.04 (1.01 ~ 1.08)	**0.007**
Pain-to-balloon time	1.20 (1.09 ~ 1.31)	**<0.001**	1.20 (1.09 ~ 1.33)	**<0.001**
RBC	1.87 (1.09 ~ 3.22)	**0.024**	1.85 (1.02 ~ 3.35)	**0.043**
Leukocytes	1.19 (1.07 ~ 1.34)	**0.002**		
Neutrophils	1.23 (1.10 ~ 1.39)	**<0.001**		
NLR	1.21 (1.10 ~ 1.35)	**<0.001**	1.23 (1.09 ~ 1.39)	**<0.001**
CRP	1.02 (1.01 ~ 1.03)	**0.009**	1.02 (1.01 ~ 1.03)	**0.03**
TG	1.63 (1.19 ~ 2.23)	**0.002**		
Glucose	1.17 (1.06 ~ 1.30)	**0.002**		
HbA1c	1.47 (1.16 ~ 1.85)	**0.001**		
TyG Index	3.36 (1.96 ~ 5.76)	**<0.001**	2.98 (1.69 ~ 5.27)	**<0.001**

OR, Odds Ratio; CI, Confidence Interval; NLR, neutrophil-to-lymphocyte ratio; CRP, C-reactive protein; TG, triglyceride; HbA1c, glycated hemoglobin; TyG, triglyceride-glucose. Variables with *P* < 0.05 in univariate analysis entered multivariable regression. A bidirectional stepwise method was used. We assessed multicollinearity using VIF (threshold <5). Leukocytes, Neutrophils, TG and glucose were excluded due to VIF > 5. This improved model stability. The final model retained independent predictors with minimal collinearity.

### Stratification by NLR and TyG and clinical outcomes

**Fig 3:**
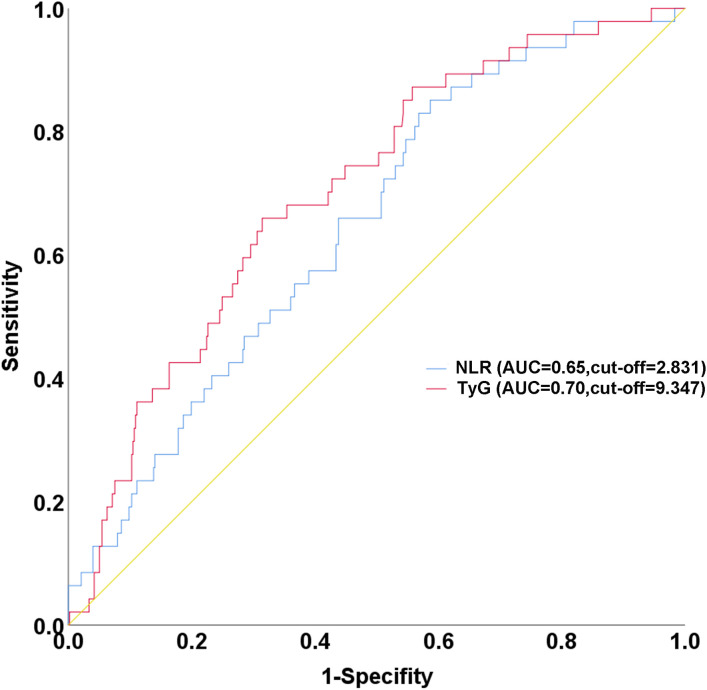
Receiver operating characteristic (ROC) curves of the NLR and TyG for predicting the no-reflow phenomenon. The TyG index (red line) demonstrated an area under the curve (AUC) of 0.70 (95% CI: 0.63-0.78), with an optimal cut-off value of 9.347. The NLR (blue line) yielded an AUC of 0.65 (95% CI: 0.57-0.72), with an optimal cut-off of 2.831. NLR, neutrophil-to-lymphocyte ratio; TyG, triglyceride-glucose.

ROC curve analysis demonstrated significant predictive value for both NLR (AUC 0.65, 95% CI 0.57–0.72) and TyG index (AUC 0.70, 95% CI 0.63–0.78) in identifying the no-reflow phenomenon (Fig 3). Using the optimal cut-off values (NLR = 2.831, TyG = 9.347) derived from this analysis, patients were stratified into four groups to assess the individual and combined influence of inflammatory and metabolic dysregulation (Table 3). Baseline demographic and clinical characteristics—including age, sex, cardiovascular risk factors, and pain-to-balloon time—were well balanced across the four groups (all *P* > 0.05). In contrast, the stratification markers NLR and TyG index differed significantly among groups (*P* < 0.001), confirming the validity of the grouping strategy. More importantly, a pronounced gradient in no-reflow incidence was observed across the strata (*P* < 0.001). Patients in Group 1 (Low NLR + Low TyG) had the lowest risk (1.49%), whereas those in Group 4 (High NLR + High TyG) exhibited a markedly elevated incidence of no-reflow (23.21%). Groups 2 and 3, each characterized by an isolated elevation in one marker, showed intermediate risk levels of 6.73% and 7.14%, respectively. These results highlight the synergistic impact of concurrent inflammatory and metabolic dysregulation on microvascular impairment after PPCI.

**Table 3:** Baseline characteristics stratified by NLR and TyG categories.

Characteristics	Group 1 (Low NLR + Low TyG) (n = 134)	Group 2 (High NLR + Low TyG) (n = 208)	Group 3 (Low NLR + High TyG) (n = 70)	Group 4 (High NLR + High TyG) (n = 112)	*P*-value
Age, years	59.86 ± 12.52	60.86 ± 10.92	60.83 ± 12.68	61.98 ± 11.43	0.57
Male, n(%)	100 (74.63)	148 (71.15)	54 (77.14)	85 (75.89)	0.692
Hypertension, n(%)	78 (58.21)	111 (53.37)	42 (60.00)	67 (59.82)	0.611
Hyperlipidemia, n(%)	36 (26.87)	57 (27.40)	13 (18.57)	26 (23.21)	0.458
Smoking, n(%)	40 (29.85)	74 (35.58)	25 (35.71)	45 (40.18)	0.404
Pain-to-balloon (hour)	6.28 ± 3.46	6.54 ± 3.18	7.16 ± 3.55	6.76 ± 3.40	0.329
NLR	2.26 (1.95,2.54)	4.24 (3.45,5.43)	2.18 (1.90,2.55)	3.99 (3.25,5.54)	**<0.001**
TyG index	8.84 (8.56,9.09)	8.86 (8.65,9.06)	9.68 (9.52,9.97)	9.68 (9.53,10.02)	**<0.001**
No-reflow, n(%)	2 (1.49)	14 (6.73)	5 (7.14)	26 (23.21)	**<0.001**

Data are presented as mean ± standard deviation, median [interquartile range], or n(%). Group 1: NLR < 2.831 and TyG < 9.347; Group 2: NLR ≥ 2.831 and TyG < 9.347; Group 3: NLR < 2.831 and TyG ≥ 9.347; Group 4: NLR ≥ 2.831 and TyG ≥ 9.347. *P*-values were derived from ANOVA, Kruskal-Wallis test, or Chi-square test as appropriate.

### NLR-TyG risk stratification independently predicts no-reflow in multivariable analysis

After adjustment for age and pain-to-balloon time in the multivariable logistic regression model, the NLR-TyG risk stratification remained a significant independent predictor of no-reflow (Table 4). A clear risk gradient was maintained across the stratified groups, with Group 4 (High NLR + High TyG) demonstrating substantially elevated odds (adjusted OR 19.23, 95% CI 4.35–83.33) compared to Group 1 (reference). Patients with isolated elevations—Group 2 (High NLR only) and Group 3 (High TyG only)—showed intermediate risk levels, with adjusted ORs of 4.15 and 4.48, respectively. In the final model, both increasing age (adjusted OR 1.04 per year) and prolonged pain-to-balloon time (adjusted OR 1.21 per hour) also remained statistically significant independent predictors.

**Table 4:** Association of NLR-TyG Risk Stratification with No-Reflow Phenomenon in Multivariable Analysis.

Variables	Adjusted OR (95% CI)	*P-*value
**Risk Stratification**		
Group 1 (Low NLR + Low TyG)	Reference	
Group 2 (High NLR + Low TyG)	4.15 (2.02 ~ 8.55)	**<0.001**
Group 3 (Low NLR + High TyG)	4.48 (1.58 ~ 12.66)	**0.005**
Group 4 (High NLR + High TyG)	19.23 (4.35 ~ 83.33)	**<0.001**
**Other Variables**		
Age (per 1-year increase)	1.04(1.00 ~ 1.07)	**0.024**
Pain-to-balloon time (per 1-hour increase)	1.21 (1.10 ~ 1.33)	**<0.001**

The multivariable logistic regression model demonstrates the independent association between NLR-TyG risk stratification and no-reflow phenomenon after adjustment for age and pain-to-balloon time. CI, confidence interval; NLR, neutrophil-to-lymphocyte ratio; OR, odds ratio; TyG, triglyceride-glucose index.

### Incremental predictive value of NLR and TyG

**Fig 4:**
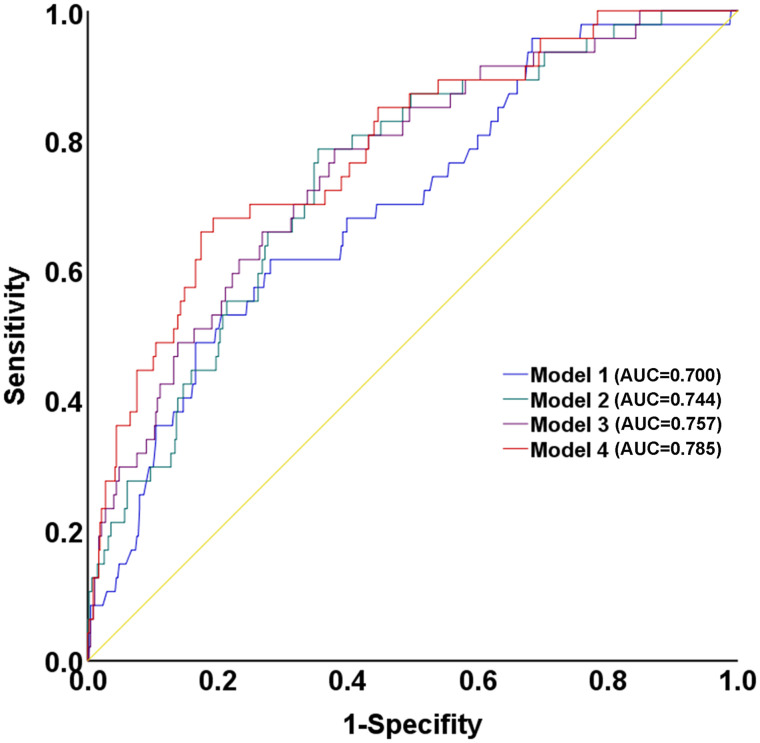
Receiver operating characteristic (ROC) curves of sequential multivariable models for predicting the no-reflow phenomenon. The baseline model of age and pain-to-balloon time (Model 1, AUC = 0.700) was incrementally improved by adding NLR (Model 2, AUC = 0.744) and the TyG index (Model 3, AUC = 0.757). The full model (Model 4), containing all predictors, yielded the highest AUC of 0.785. Model 1:age and pain-to-balloon time; Model 2: Model 1 + NLR; Model 3: Model 1 + TyG；Model 4: Model 1 + NLR + TyG.

**Fig 5:**
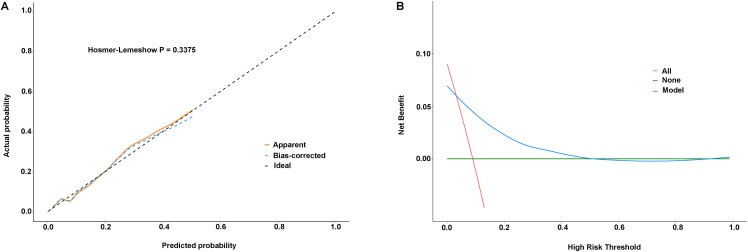
Calibration and decision curve analyses for the final model (Model 4). Model 4 included four predictors: age, pain-to-balloon time, neutrophil-to-lymphocyte ratio (NLR), and triglyceride-glucose (TyG) index. **(A)** Calibration plot. The solid line shows good agreement between predicted and observed probabilities of no-reflow, indicating good calibration. **(B)** Decision curve analysis. The model provides positive net benefit across threshold probabilities of 0–10%, outperforming both “treat all” and “treat none” strategies, supporting its clinical utility.

The sequential addition of NLR and TyG index to the baseline model (Model 1) containing age and pain-to-balloon time resulted in progressively improved discriminative ability for predicting no-reflow (Table 5 and Fig 4). The baseline model achieved an AUC of 0.700 (95% CI: 0.658–0.739). Incorporating NLR alone (Model 2) increased the AUC to 0.744, while adding TyG index alone (Model 3) yielded an AUC of 0.757. The full model (Model 4) combining both NLR and TyG index demonstrated the highest predictive performance with an AUC of 0.785 (95% CI: 0.748–0.820). This stepwise improvement suggests that NLR and the TyG index capture complementary pathophysiological pathways, with their combination yielding the most robust predictive performance. Bootstrap internal validation with 1,000 resamples confirmed the stability of the final model (Model 4: age, pain-to-balloon time, NLR, and TyG index). The bootstrap-derived 95% BCa confidence interval for the AUC was 0.699–0.849, which contained the original point estimate of 0.785, indicating minimal overfitting. The Hosmer-Lemeshow test was non-significant (χ² = 9.057, *P* = 0.338), indicating good calibration (Fig 5A). Decision curve analysis demonstrated that the model provided positive net benefit across threshold probabilities of 0–10%, outperforming both the “treat all” and “treat none” strategies (Fig 5B), indicating clinical utility of NLR and TyG index for guiding clinical decisions.

**Table 5:** Comparison of predictive performance across sequential multivariable Models for no-reflow phenomenon.

	Model Components	AUC (95% CI)	ΔAUC vs. Model 1	*P*-Value for ΔAUC
Model 1	Age + Pain-to-balloon time	0.700 (0.658-0.739)		
Model 2	Model 1 + NLR	0.744 (0.704-0.780)	0.044	0.056
Model 3	Model 1 + TyG	0.757 (0.718-0.793)	0.057	0.016
Model 4	Model 1 + NLR + TyG	0.785 (0.748-0.820)	0.085	0.003

AUC, area under the curve; CI, confidence interval; NLR, neutrophil-to-lymphocyte ratio; TyG, triglyceride-glucose. The ΔAUC and P values result from pairwise comparison with Model 1 using DeLong’s test.

### Subgroup analysis and interaction tests

Subgroup analyses stratified by sex, age, and BMI demonstrated consistent predictive effects of TyG index and NLR across different patient populations (Table 6). Although TyG and NLR did not reach statistical significance in females and normal-weight individuals, interaction tests revealed no significant effect modification by sex, age, or BMI (all *P* for interaction > 0.05). The lack of significance in these subgroups is likely attributable to smaller sample sizes and limited statistical power rather than true heterogeneity. These findings support the robustness and generalizability of TyG index and NLR as predictors of no-reflow in T2DM patients with STEMI.

**Table 6:** Subgroup analysis and interaction tests for the association of TyG Index and NLR with no-reflow.

Subgroup	No. of patients	No-Reflow Events n (%)	TyG Index	NLR	*P* for interaction
			OR (95% CI)	OR (95% CI)	TyG/ NLR
Overall	524	47 (8.97)	2.98 (1.69-5.27)	1.23 (1.09-1.39)	
Sex					0.103/ 0.208
Male	387	34 (8.79)	4.25 (2.14-8.45)	1.31 (1.14-1.51)	
Female	137	13 (9.49)	1.45 (0.47-4.47)	1.06 (0.78-1.44)	
Age					0.111/ 0.930
<60 years	225	12 (5.33)	6.84 (2.01-23.29)	1.24 (1.02-1.52)	
≥60 years	299	35 (11.71)	2.28 (1.21-4.29)	1.25 (1.08-1.45)	
BMI					0.735/ 0.353
<25 kg/m²	198	14 (7.07)	3.64 (1.23-10.75)	1.16 (0.91-1.49)	
≥25 kg/m²	326	33 (10.12)	2.89 (1.48-5.66)	1.28 (1.12-1.47)	

OR: odds ratio; CI: confidence interval; TyG, triglyceride-glucose; NLR, neutrophil-to-lymphocyte ratio; BMI, body mass index.

## Discussion

The present study investigated the predictive value of inflammatory and metabolic biomarkers for the no-reflow phenomenon following PPCI in a high-risk population of STEMI patients with comorbid T2DM. Our principal findings demonstrate that both NLR and TyG index serve as independent predictors of no-reflow. A risk stratification model constructed from these two markers revealed a pronounced gradient in risk across the four defined subgroups, with the dual-high-risk group exhibiting a marked escalation in susceptibility. Furthermore, the incorporation of NLR and the TyG index into a baseline clinical model significantly enhanced its predictive performance for no-reflow. Collectively, these findings underscore that a combined assessment of inflammatory (NLR) and metabolic (TyG) status provides a powerful tool for the early identification of high-risk patients.

NLR serves as an integrated marker of systemic inflammatory burden that reflects two distinct immune pathways. This readily available hematological parameter has emerged as a practical tool for assessing inflammatory status in cardiovascular diseases. Evidence from previous studies strongly supports its prognostic value, with an elevated NLR being linked to more extensive coronary artery disease, a higher risk of heart failure, increased mortality, and a greater incidence of the no-reflow phenomenon in STEMI patients [16–20]. Some studies have reported only modest diagnostic performance when it is used in isolation to predict no-reflow [21]. Our study extends this evidence to STEMI patients with T2DM, a group highly vulnerable to microvascular injury due to chronic inflammation. In our cohort, multivariable logistic regression confirmed NLR as an independent predictor of no-reflow (adjusted OR 1.23, 95% CI 1.09–1.39, *P* < 0.001). This finding confirms that inflammatory pathways, as captured by this marker, contribute significantly to microvascular injury in T2DM-STEMI patients, independent of metabolic status and other clinical confounders.

IR individuals are prone to develop various metabolic abnormalities such as hyperglycemia, dyslipidemia, and hypertension, all of which are intricately linked to unfavorable cardiovascular disease outcomes [22,23]. TyG index has emerged as a reliable and effective measure for identifying IR [24]. Previous investigations have demonstrated its utility as a robust marker for risk stratification and prognosis assessment in patients with acute coronary syndromes (ACS), irrespective of diabetic status [25–28]. Previous studies have established an independent association between the TyG index and the no-reflow phenomenon in STEMI patients with diabetes mellitus [29]. Our analysis identified the TyG index as the strongest independent predictor for no-reflow in this high-risk population (adjusted OR 2.98, 95% CI 1.69–5.27, *P* < 0.001). This underscores that the profound metabolic dysregulation represented by the TyG index provides crucial prognostic information over and above traditional risk factors.

The negligible correlation between NLR and the TyG index (r = 0.050, *P* = 0.250) statistically confirms their capture of fundamentally distinct pathophysiological processes. This finding underscores their distinct pathophysiological origins: the NLR primarily reflects the state of systemic inflammatory activation, whereas the TyG index represents the degree of metabolic dysregulation. By capturing these two complementary yet independent pathways, their integration provides a more comprehensive risk assessment for no-reflow, which explains their superior predictive power when used in combination compared to either marker alone.

This biological independence was powerfully translated into clinical risk stratification. When patients were stratified using the optimal cut-offs (NLR = 2.831, TyG = 9.347), a dramatic risk gradient was revealed. The no-reflow incidence increased sharply from 1.49% in Group 1 (low-risk) to 23.21% in Group 4 (dual-high-risk), demonstrating exceptional discriminatory power. Critically, we observed a synergistic rather than simply additive effect. The risk in Group 4 substantially exceeded the combined individual risks of Groups 2 and 3 (6.73% and 7.14%, respectively). This profound risk amplification indicates that in diabetic patients, inflammatory and metabolic disturbances engage in deep pathophysiological cross-talk, jointly driving microvascular injury to its maximum potential. The clinical utility of this model is immediately apparent. Utilizing routinely available laboratory parameters, this economical stratification enables rapid identification of “dual-high-risk” patients during the PPCI perioperative period. These patients represent a critical subgroup that may benefit most from intensified monitoring and consideration of adjunctive therapeutic strategies aimed at microvascular protection.

The prompt identification of STEMI patients at risk for the no-reflow phenomenon remains a significant clinical challenge [30]. Hematological biomarkers are objective, readily quantifiable proteins that reflect diverse pathophysiological pathways and can provide biologically derived prognostic information beyond traditional clinical risk factors [31–34]. This approach is supported by numerous previous studies investigating combined biomarkers for risk stratification in myocardial infarction. The sequential comparison of our prediction models demonstrates the incremental value of adding these biomarkers. The improvement in AUC from Model 1 (0.700) to Model 4 (0.785) demonstrates that NLR and TyG index provide additional predictive information beyond conventional clinical factors. Notably, the combination of both markers yielded the highest predictive performance (AUC 0.785), suggesting that inflammatory and metabolic pathways capture complementary aspects of no-reflow pathophysiology. Although the addition of NLR to the TyG-only model did not reach statistical significance in our cohort, the observed trend is consistent with the biological interplay between inflammation and insulin resistance. Therefore, beginning with a foundational clinical model, we have developed an integrated approach that captures both the metabolic and inflammatory aspects of no-reflow pathophysiology.

The consistency of our findings across different patient populations was further supported by subgroup and interaction analyses. Although TyG index and NLR did not reach statistical significance in females and normal-weight individuals, the interaction tests revealed no significant effect modification by sex, age, or BMI (all *P* for interaction > 0.05). These results suggest that the observed differences between subgroups are likely attributable to limited statistical power in smaller subgroups (females: n = 137, 13 events; normal-weight: n = 198, 14 events) rather than true heterogeneity of effects. The absence of significant interactions indicates that the predictive value of TyG index and NLR for no-reflow is consistent across diverse patient populations, supporting the broad applicability of the NLR-TyG risk stratification tool in clinical practice.

## Study limitations

Several limitations of our study should be acknowledged. First, this was a single-center, retrospective investigation, which may introduce selection bias and limit the generalizability of our findings. Consequently, our conclusions require validation in prospective, multi-center studies. Second, the absolute number of no-reflow events (n = 47) was relatively modest, yielding an events-per-variable ratio of approximately 7.83. However, bootstrap validation with 1,000 resamples confirmed model stability and minimal overfitting. Nevertheless, the modest event number may constrain our ability to explore additional predictors, and validation in larger cohorts is warranted. Third, the definition of no-reflow in this study was based solely on post-procedural TIMI flow grade. We acknowledge that more comprehensive perfusion parameters, such as myocardial blush grade (MBG) or the degree of ST-segment resolution (STR), are widely accepted indicators of microvascular reperfusion and provide a more nuanced assessment of no-reflow. The absence of these parameters limits the granularity of our evaluation of microvascular injury. Fourth, both NLR and TyG index were measured only once at admission, failing to capture their dynamic changes during hospitalization. Additionally, their optimal cut-off values were derived from ROC analysis within the same cohort, which may introduce optimism bias and require external validation. Finally, despite comprehensive multivariate adjustments, we cannot completely rule out residual confounding from unmeasured factors, such as glycemic variability or specific hypoglycemic regimens.

## Conclusion

This study establishes that combining NLR and TyG index effectively identifies T2DM-STEMI patients at highest risk for no-reflow. Integrating these readily available biomarkers into clinical practice could guide intensive perioperative management for dual-high-risk patients. Future work should focus on multicenter validation, development of an integrated risk score, and assessing whether targeted interventions improve outcomes in this population.

## Supporting information

S1 File Data_anonymized. (XLSX)
