# Correction: The triglyceride-glucose index and neutrophil-to-lymphocyte ratio jointly predict the no-reflow phenomenon in T2DM patients with STEMI after primary PCI

**DOI:** 10.1371/journal.pone.0361392

**Published:** 2026-10-23

**Authors:** The PLOS One Staff

The first author, Jingyan Yang, is incorrectly noted as the corresponding author. The correct corresponding author is Juan Zhang. Juan Zhang’s email address is: awwa6940@sina.com.

The publisher apologizes for the error.
